# Camptothecin sensitizes androgen-independent prostate cancer cells to anti-Fas-induced apoptosis

**DOI:** 10.1038/sj.bjc.6690365

**Published:** 1999-05-01

**Authors:** A P Costa-Pereira, T G Cotter

**Affiliations:** Tumour Biology Laboratory, Department of Biochemistry, University College, Ireland

**Keywords:** apoptosis, prostate cancer, Fas, caspases, reactive oxygen intermediates

## Abstract

Despite expressing both Fas and Fas ligand, DU145 and LNCaP prostate cancer cells were resistant to anti-Fas-induced cell death. Resistance to Fas-mediated cytotoxicity could be overcome in DU145, but not in LNCaP, cells by pretreating cells with sublethal doses of cytotoxic drugs, such as camptothecin. Activated caspases were shown to be required for this cytotoxicity. Indeed, poly(ADP-Ribose) polymerase was shown to be proteolytically cleaved in cells treated with camptothecin plus anti-Fas, but not in cells treated with anti-Fas only. Moreover, pretreatment of cells with ZVAD completely blocked camptothecin-mediated Fas-induced apoptosis. Sensitization of cells to Fas-induced cell death did not involve up-regulation of Fas or FasL, and it was independent of alterations in the cell cycle. Reactive oxygen intermediates (ROI) have been shown to be important mediators of drug-induced apoptosis. Here, we demonstrate that treatment of DU145 cells with camptothecin, anti-Fas, or both, did not alter the intracellular levels of peroxide or superoxide anion. © 1999 Cancer Research Campaign


*British Journal of Cancer* (1999) **80**(3/4), 371–378
© 1999 Cancer Research Campaign
Article no. bjoc.1998.0365

### Camptothecin sensitizes androgen-independent prostate cancer cells to anti-Fas-induced apoptosis

**AP Costa-Pereira and TG Cotter**

Tumour Biology Laboratory, Department of Biochemistry, University College, Ireland

**Summary** Despite expressing both Fas and Fas ligand, DU145 and LNCaP prostate cancer cells were resistant to anti-Fas-induced cell death. Resistance to Fas-mediated cytotoxicity could be overcome in DU145, but not in LNCaP, cells by pretreating cells with sublethal doses of cytotoxic drugs, such as camptothecin. Activated caspases were shown to be required for this cytotoxicity. Indeed, poly(ADP-Ribose) polymerase was shown to be proteolytically cleaved in cells treated with camptothecin plus anti-Fas, but not in cells treated with anti-Fas only. Moreover, pretreatment of cells with ZVAD completely blocked camptothecin-mediated Fas-induced apoptosis. Sensitization of cells to Fas-induced cell death did not involve up-regulation of Fas or FasL, and it was independent of alterations in the cell cycle. Reactive oxygen intermediates (ROI) have been shown to be important mediators of drug-induced apoptosis. Here, we demonstrate that treatment of DU145 cells with camptothecin, anti-Fas, or both, did not alter the intracellular levels of peroxide or superoxide anion.

**Keywords:** apoptosis; prostate cancer; Fas; caspases; reactive oxygen intermediates

The prostate in ageing males is prone to benign and malignant proliferative changes (Carter and Coffey, 1990). Indeed, cancer of the prostate is the most prevalent malignancy and the second-highest fatal neoplasm among Western males (Chung, 1995). The mechanism of prostatic carcinogenesis and tumour development is likely to involve a multistep progression from premalignant cells to cancerous cells which proliferate and metastasize (Huggins and Hodges, 1941). Initially, the growth and development of prostate cancer appears to be androgen-dependent. Nearly all men with metastatic prostate cancer, treated with surgical or chemical castration, have an initial beneficial response to androgen withdrawal, which induces tumour regression by apoptosis. Although this initial approach has significant palliative value, nearly all patients relapse with a hormonally independent disease (Denmeade et al, 1996). This hormonally independent disease also becomes resistant to additional therapies, such as chemotherapy (Yagoda and Petrylak, 1992), and displays growth autonomy (Figueroa et al, 1995). In the normal prostatic epithelium, cell proliferation is balanced by an equal rate of apoptotic cell death. In prostate cancer, however, this fine balance is lost, causing increased cellular mass and tumour progression (Denmeade et al, 1996). Hence, an effective therapy for prostate cancer should redress the imbalance in programmed cell death, which seems to be important for disease progression.

The Fas (APO-1/CD95) receptor belongs to the nerve growth factor/tumour necrosis factor (NGF/TNF) receptor family and mediates apoptosis in a variety of cell types (Yonehara et al, 1989; Oehm et al, 1992). Although Fas seems to be a key regulator of apoptosis, the pathways through which this molecule exerts its actions and the mechanisms which determine whether any given cell is sensitive or resistant to Fas remain to be fully elucidated. It seems that there is not a strict correlation between Fas and Fas ligand (FasL) expression and sensitivity to Fas-mediated cytotoxicity. In fact, activation of the Fas signalling pathway(s) in a variety of tumour cell lines and in activated lymphocytes does not always trigger apoptosis (Owen-Schaub et al, 1994). Caspases are thought to play a central role in apoptosis and de-regulation of some caspases may contribute to the development of some cancers. Furthermore, there is evidence that caspase-dependent cleavage of specific proteins may abrogate important survival pathways (Widmann et al, 1998). Importantly, commitment to Fas-induced apoptosis was shown to require caspase activity (Longthorne et al, 1997). Following Fas trimerization there seems to be sequential activation of caspase-1 (ICE)- and caspase-3 (CPP32)-like proteases (Enari et al, 1996). The activated caspases can then cleave various substrates, which include Poly(ADP-Ribose) polymerase (PARP), Lamin, rho-GDI, Actin, Ras GTPase-activating protein, Raf-1 and Akt-1, eventually resulting in morphological changes to the cells and nuclei (Nagata, 1997; Widmann et al, 1998).

Previous work from our laboratory has shown the importance of reactive oxygen intermediates (ROI) during apoptosis in HL-60 and U937 leukaemic cell lines (McGowan et al, 1994). In particular, peroxide production has been reported in response to a variety of inducers of apoptosis (Gorman et al, 1997b; McGowan et al, 1998). The production of peroxide after cytotoxic insult and the ability of antioxidants to inhibit PARP cleavage and apoptosis, suggest that early activation of caspases may act as a common response to cytotoxic insult and that ROI may serve as common mediators of cell death in leukaemic cell lines (McGowan et al, 1996). In contrast to a well-established role in chemotherapeutic drug-induced apoptosis, the involvement of ROI in Fas-mediated signal transduction pathway(s) is still controversial. Schulze-Osthoff and colleagues (1994) have shown that neither inhibitors of the mitochondrial respiratory chain nor antioxidants protected murine fibrosarcoma cells from Fas-mediated cell death. In contrast, ROI have been reported to be involved in Fas-mediated

*Received 7 August 1998*
*Revised 2 November 1998*
*Accepted 10 November 1998*

*Correspondence to:* TG Cotter signal transduction pathway and to be major mediators of apoptosis in neutrophils (Kasahara et al, 1997). In addition, Dobbelsteen and co-workers (1996) have shown that Fas ligation results in a rapid and specific efflux of glutathione (GSH). Severe depletion of GSH will lower the reducing capacity of a cell, enhancing oxidative stress independent of any increase in the production of ROI. Furthermore, an oxidative stress due to elevated intracellular superoxide levels has been shown to block Fas-mediated apoptosis in cells that were constitutively Fas-sensitive (Clément and Stamenkovic, 1996). These studies suggest that an imbalance in ROI levels may play a role in Fas-mediated apoptosis.

Due to the complexity of prostatic cancer, successful treatment of the disease is likely to require a multimodality therapy involving cytokines (immunotherapy), cytotoxic drugs (chemotherapy) and/or hormonal ablation. The present study was designed to examine possible ways of bypassing the resistance of prostate cancer cell lines to Fas-mediated apoptosis and to further elucidate the signalling pathways involved in Fas signal transduction.

### MATERIALS AND METHODS

### Reagents and cell lines

Prostate cancer cell lines LNCaP and DU145 were obtained from American Type Culture Collection (Rockville, MD, USA). Primary antibodies used in this study were anti-Fas IgM, clone Ch-11 (Upstate Biotechnology, Waltham, MA, USA); anti-Fas IgG, clone SM1/1 (Bender MedSystems, Heidelberg, Germany); anti-FasL, N-20 (Santa Cruz, CA, USA); anti-PARP, 06-557-MN (Upstate Biotechnology) and anti-β-actin (Sigma, Poole, UK). The caspase inhibitor benzyloxycarbonyl-Val-Ala-Asp-fluoromethyl ketone (ZVAD) was purchased from Enzyme System Products (Dublin, CA, USA). The fluorescent probes 2′,7′-dichlorofluorescin diacetate (DCFH-DA), dihydroethidium (DHE) and 5,5′,6,6′-tetrachloro-1,1′,3,3′-tetraethyl-benzimidazolylcarbocyanine iodide (JC-1) were obtained from Molecular Probes (Leiden, The Netherlands). Cell culture reagents were purchased from Gibco/BRL (Paisley, UK), protein gel electrophoresis reagents from BioRad (Hertfordshire, UK), cytotoxic drugs, propidium iodide (PI) and RNAase were from Sigma, and all other reagents were obtained from BDH (Cork, Ireland).

### Cell culture

Prostate cancer cells were cultured in RPMI-1640 medium containing 5% (v/v) fetal calf serum, 2 mM L-glutamine, supplemented with penicillin–streptomycin (10 IU $ml^{-1}$, 10 μg $ml^{-1}$) and fungizone amphotericin B (2.5 μg $ml^{-1}$). Cells were grown at 37°C in a humidified 5% carbon dioxide atmosphere and were routinely subcultured every 4 days. Cell density and viability were assessed by trypan blue exclusion, using a haemocytometer.

### Expression of Fas and FasL by flow cytometry

For FasL expression assessment, cells were fixed with PBS/*p*-formaldehyde (1%, w/v, 30 min, room temperature) and permeabilized with cold ethanol (70%, v/v, overnight, −20°C). Cells were incubated with a rabbit anti-FasL (2 μg $ml^{-1}$, 1 h, 4°C), followed by the secondary antibody (fluorescence isothiocyanate-conjugated, 30 min, 4°C). Cells were analysed on a FACScan (Becton Dickinson, Mountain View, CA, USA), equipped with Cell Quest software (Becton Dickinson). Fixation and permeabilization steps were omitted when analysing cells for Fas expression (mouse anti-Fas IgG used at 2 μg $ml^{-1}$).

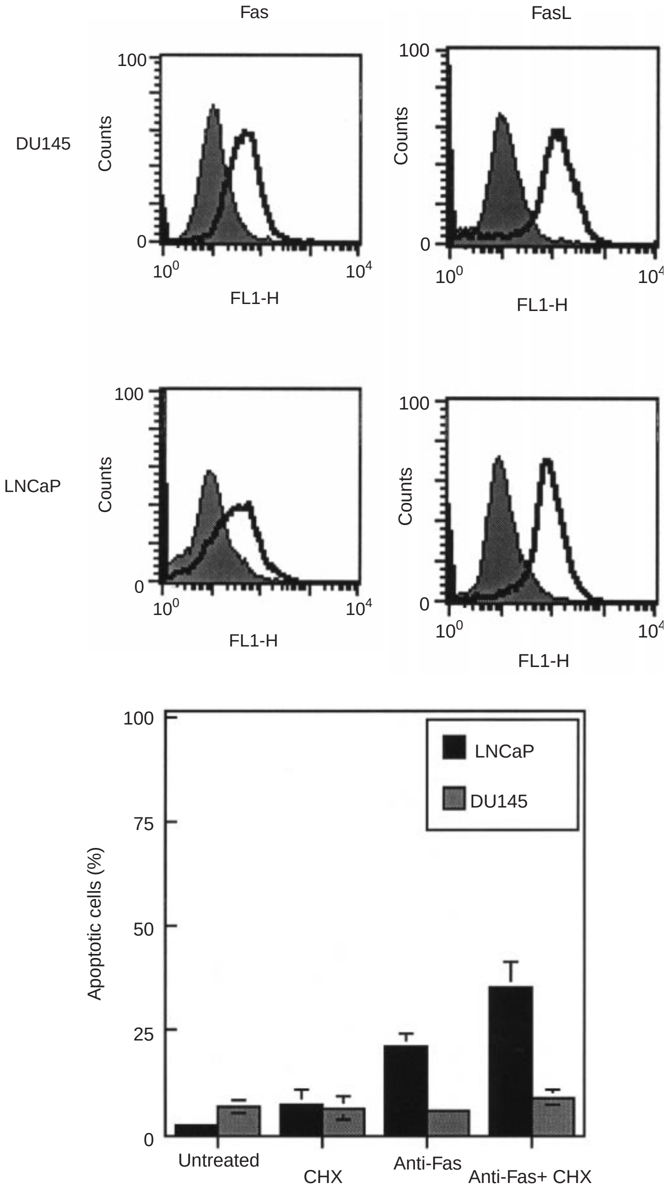

**Figure 1** (**A**) Flow cytometric analysis of Fas and FasL in prostate cancer cell lines. LNCaP and DU145 cells were stained with a FITC-conjugated secondary antibody alone (intrinsic fluorescence control), (●); or with anti-Fas or anti-FasL, followed by the relevant secondary FITC-conjugated antibody, (○). Data shown is representative of at least three independent experiments. (**B**) Fas-Induced cell death in prostate cancer cell lines. Cells were incubated with cyclohexamide (CHX, 0.25 μg $ml^{-1}$), anti-Fas IgM (200 ng $ml^{-1}$), or both for 24 h, and apoptosis levels were assessed by morphological analysis as described in Materials and Methods. The data (from three independent microscopic fields) represents mean apoptosis±standard error of the mean and results shown are representative of at least three independent experiments

### Fas-induced cell death

Cells were plated in 24-well plates and treatment with anti-Fas IgM (200 ng/ml) was carried out for 24 h. Whenever used, cytotoxic

© Cancer Research Campaign 1999

drugs (100 ng ml$^{-1}$ actinomycin D, 100 ng ml$^{-1}$ camptothecin, 1.25 μg ml$^{-1}$ etoposide, 1 μg ml$^{-1}$ *cis*-platinum(II)diaminedichloride, 0.25 μg ml$^{-1}$ cyclohexamide), were added 5 min prior to anti-Fas IgM. When required, ZVAD (50 μM) was added 5 min prior the addition of cytotoxics.

### Morphological analysis

Briefly, supernatants were transferred to labelled test tubes and cells were washed once in HBSS. Cells in each well were then trypsinized using Trypsin/EDTA (TE) for 10 min at 37°C. TE was neutralized with complete media and cells were transferred to the appropriate test tube. Cells were then cytospun at 100 ***g*** (500 rpm) for 2 min. Slides were stained using the Rapi-Diff II kit (Langan Back Services, Ireland) and cell morphology was assessed by light microscopy, using previously defined criteria (Wyllie et al, 1980).

### Cell cycle analysis

Freshly harvested cells (2.5 × 10$^5$ ml$^{-1}$) were fixed in ice-cold ethanol (70%, v/v), on ice for 15 min. Ethanol was removed by centrifugation (200 g × 5 min) and cells were resuspended in 1 ml of a PBS/PI (50 μg ml$^{-1}$) solution containing RNAase (0.5 mg ml$^{-1}$). Following a 30 min incubation on ice, in the dark, cells were analysed by flow cytometry in FL2-H, on a FACScan equipped with Cell Quest software.

### Western blot

Whole cell lysates were prepared by solubilizing ~10$^6$ cells in 20 μl of lysing buffer (10 mM Hepes/NaOH, pH 8.0; 150 mM sodium chloride; 500 mM sucrose; 1 mM EDTA; 0.15 mM spermine; 1.0% v/v Triton X-100; 0.2 mM phenylmethylsulphonyl fluoride; 1.0 μg ml$^{-1}$ antipain; 1.0 μg ml$^{-1}$ aprotinin; 1.0 μg ml$^{-1}$ chymostatin; 0.1 μg ml$^{-1}$ leupeptin; 4.0 μg ml$^{-1}$ pepstatin) and incubating cells on ice for 1 h. Prior to electrophoresis, cells were diluted in 2× sodium dodecyl sulphate (SDS) gel-loading buffer (100 mM Tris–HCl, pH 6.8; 200 mM dithiothreitol; 4.0% w/v SDS; 0.2% w/v bromophenol blue; 20% v/v glycerol) and boiled for 15 min. Proteins were separated in a 10% (w/v) polyacrylamide gel and blotted onto Trans-Blot nitrocellulose membrane. After blocking, the membrane was incubated with anti-human PARP (1/500 dilution, 4°C, overnight), followed by an anti-rabbit-HRP-conjugated antibody (1/1000, 1 h, room temperature). The membrane was probed with ECL (Amersham) and exposed to Kodak X-OMAT LS film (Sigma).

### Detection of intracellular ROI and measurement of mitochondrial transmembrane potential (Δψm)

Mitochondrial membrane depolarization, peroxide and superoxide levels were measured as previously described (Gorman et al, 1997a; Costa-Pereira and Cotter, 1999). Briefly, cells (2.5 × 10$^5$) were harvested and incubated with 5 μg ml$^{-1}$ JC-1 (Δψm measurement), for 15 min; or with 5 μM DCFH-DA (peroxide level) for 1 h at 37°C; or with 5 μM DHE (superoxide anion) for 30 min at 37°C. Fluorescence due to JC-1 and oxidized DCFH-DA was measured on a FACScan (Becton Dickinson, Mountain View, CA, USA), equipped with Cell Quest software (Becton Dickinson), in FL-1, whereas the fluorescence due to oxidation of DHE was measured in FL-2. Cells were treated with the apoptosis-inducing agents either before or during the incubation period, depending on the time point at which ROI or Δψm were to be studied.

### RESULTS

### Prostate cancer cells are resistant to Fas-mediated cytotoxicity

Fas and FasL expression were assessed in LNCaP and in DU145 prostate cancer cells by flow cytometry (Figure 1A). Although both cell lines expressed the ligand and the receptor, treatment of cells with anti-Fas IgM (200 ng ml$^{-1}$; 24 h) induced only moderate levels of apoptosis in the androgen-responsive cell line, LNCaP, having no significant cytotoxic effects on the androgen-independent cell line, DU145 (Figure 1B). LNCaP (but not DU145) cells were more susceptible to Fas-mediated apoptosis when treated concomitantly with anti-Fas and cyclohexamide (CHX, 0.25 μg ml$^{-1}$), suggesting the presence of an endogenous protein inhibitor of the Fas signalling pathway in these cells. These results are in contrast to an article by Rokhlin et al (1997). However, their DU145 were only susceptible to treatment with 1 μg ml$^{-1}$ anti-Fas at high concentrations of CHX (2.5–25 μg ml$^{-1}$), and the resistance displayed by their LNCaP cells is uncharacteristic of our cell lines. The sensitization of LNCaP cells mediated by CHX was not due to up-regulation of Fas, as assessed by flow cytometry (results not shown). Dose–response curves for both LNCaP and DU145 cell lines treated with anti-Fas IgM showed that, even at high anti-Fas IgM concentrations (1.5 μg ml$^{-1}$), DU145 cells were resistant to anti-Fas-induced apoptosis, indicating that there is a bona fide blockage in the Fas apoptotic pathway(s) in these cells. LNCaP cells were more susceptible to anti-Fas and there was, to a certain extent, a dependency on the dose of antibody used. The apoptosis levels, however, never surpassed those observed when LNCaP cells were treated with 0.25 μg ml$^{-1}$ CHX (data not shown).

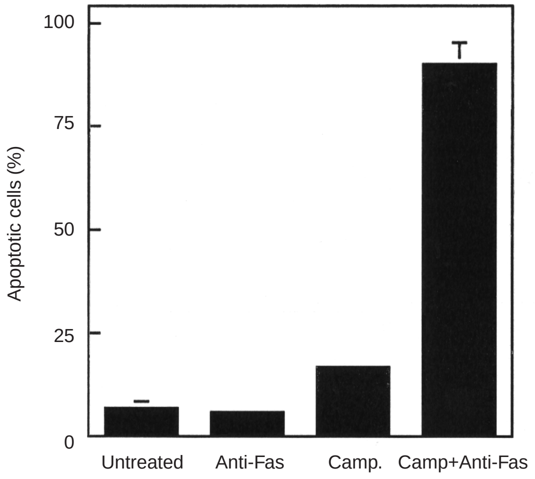

**Figure 2** Chemosensitization of androgen-independent prostate cancer cells to Fas-induced cell death. DU145 cells were treated with subtoxic doses of camptothecin (100 ng ml$^{-1}$) and concomitantly incubated with anti-Fas IgM (200 ng ml$^{-1}$), for 24 h. Apoptosis was assessed as described in Figure 1B

© Cancer Research Campaign 1999

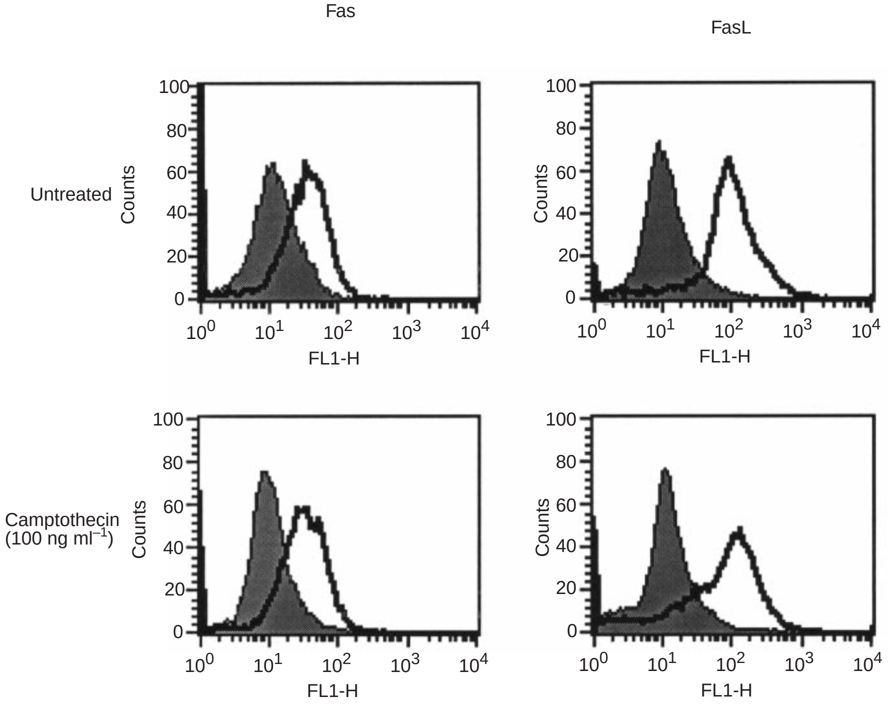

**Figure 3** Fas and FasL expression in untreated DU145 cells and in cells treated with camptothecin (100 ng ml$^{-1}$). DU145 cells were stained with a FITC-conjugated secondary antibody alone (intrinsic fluorescence control), (●); or with anti-Fas or anti-FasL, followed by the relevant secondary FITC-conjugated antibody, (○). Data are for the 24 h time point and are similar to those seen for the 4 and 8 h points

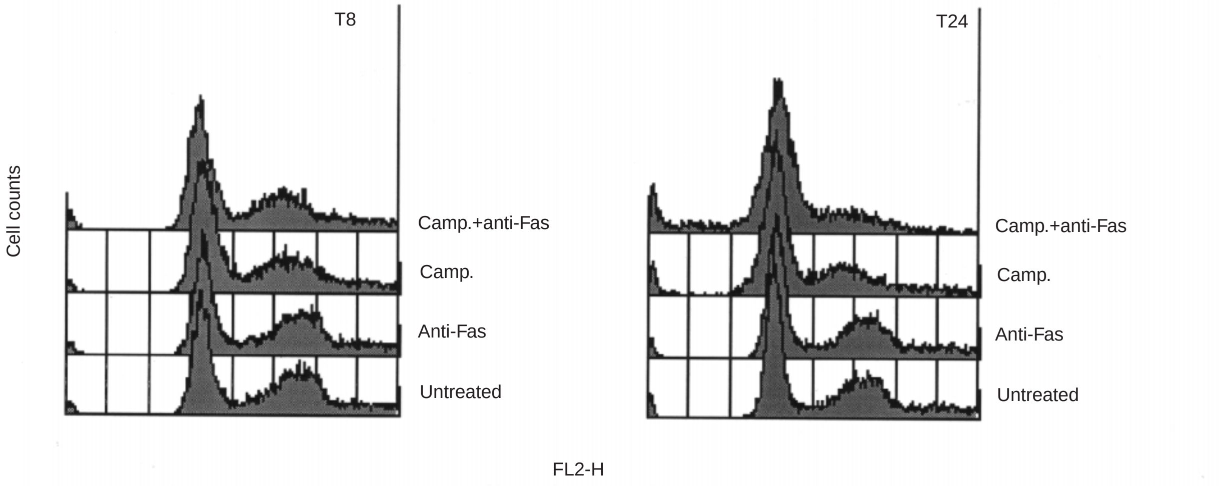

**Figure 4** Cell cycle analysis of untreated DU145 cells and cells treated with anti-Fas (200 ng ml$^{-1}$), camptothecin (100 ng ml$^{-1}$), or both, at the indicated times. The first peak represents G0/G1 phase, the second peak cells in G2/M

### Sensitization of androgen-independent prostate cancer cells to anti-Fas-induced cell death

To address the possible mechanism(s) involved in the resistance displayed by prostate cancer cells to Fas-induced apoptosis, we have used a number of cytotoxic drugs known to have distinct intracellular targets. The chemotherapeutic drugs used were: actinomycin D (RNA transcription inhibitor, which can also induce topoisomerase I and II cleavable complexes), camptothecin (topoisomerase I inhibitor), *cis*-platinum(II)diamminedichloride (CPDD) (non-classical alkylating agent, which forms intrastrands linkages with DNA) and etoposide (topoisomerase II inhibitor). DU145 were sensitized by ~20-fold (average of five independent experiments yielding similar results) when treated with subtoxic concentrations of camptothecin (100 ng ml$^{-1}$) and subsequently treated with anti-Fas IgM (200 ng ml$^{-1}$) (Figure 2). The sensitization does not seem to be particular to topoisomerase I inhibitors, as actinomycin D (100 ng ml$^{-1}$), CPDD (1.0 µg ml$^{-1}$) and etoposide (1.25 µg ml$^{-1}$) also sensitized DU145, although to a lesser extent

 *© Cancer Research Campaign 1999*

A

B

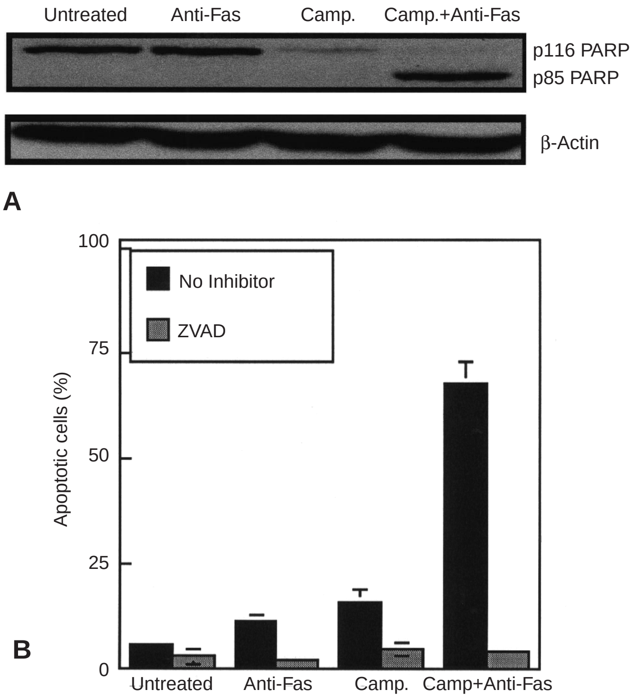

**Figure 5** (**A**) Caspases are involved in camptothecin-mediated anti-Fas-induced apoptosis of DU145 cells. Western blot analysis of PARP cleavage in untreated (lane 1) DU145 cells, or treated with anti-Fas IgM, 200 ng $ml^{-1}$ (lane 2); Camp, 100 ng $ml^{-1}$ (lane 3); Camp. and anti-Fas (lane 4). The membrane was also probed with anti-β-actin to ensure equal protein loading (30 μg protein/lane). (**B**) Inhibition of Fas-induced apoptosis in DU145 cells pretreated with camptothecin by a caspase inhibitor, ZVAD. Cells ($5 \times 10^4$ $ml^{-1}$) were seeded in 24-well plates and cultured for 48 h. Cells were incubated for 5 min with 50 μM of ZVAD, followed by treatment with camptothecin (100 ng $ml^{-1}$), anti-Fas IgM (200 ng $ml^{-1}$), or both, for 24 h. Apoptosis levels were assessed as described in Figure 1B

(data not shown). Identical concentrations of actinomycin D, camptothecin, or etoposide failed to sensitize the androgen-responsive cells, LNCaP (not shown).

### Camptothecin-mediated sensitization of DU145 cells to anti-Fas-induced cell death does not involve up-regulation of Fas or FasL proteins

Recent reports (Friesen et al, 1996; Herr et al, 1997) suggested that apoptosis caused by certain anticancer drugs was mediated by the Fas/FasL system. In order to determine whether camptothecin was sensitizing DU145 cells to Fas-mediated apoptosis by up-regulating Fas and/or FasL, cells were treated with camptothecin and flow cytometric analysis of Fas and FasL expression was assessed at different time points (4 and 24 h post-treatment). Results show that there was no change, at the protein level, of Fas or FasL (Figure 3), suggesting that sensitization was not due to upregulation of Fas and/or FasL.

### Camptothecin's effect is independent of alterations in the cell cycle

Camptothecin has been reported to cause a cell cycle arrest in S/G2 (Hsiang et al, 1989). Thus it was considered that the sensitization to Fas-mediated apoptosis could be a consequence of cell cycle arrest. Flow cytometric analysis of untreated cells, or cells treated with camptothecin (100 ng $ml^{-1}$), anti-Fas IgM (200 ng $ml^{-1}$), or camptothecin plus anti-Fas, for 8 h, showed no significant cell cycle abnormalities in the cell cycle profile of each sample. After a 24 h treatment with camptothecin plus anti-Fas IgM, only sub-G0/G1 population was slightly increased indicative of apoptosis (Figure 4).

### Activation of caspases is required for camptothecin-mediated sensitization of DU145 cells to anti-Fas-induced apoptosis

There have been several reports showing that caspases play a pivotal role in Fas-induced apoptosis (Enari et al, 1996; Longthorne and Williams, 1997). Western blot analysis demonstrated that poly(ADP-ribose) polymerase was proteolytically cleaved in camptothecin-treated cells, but not in cells treated with anti-Fas only (Figure 5A). A down-regulation of PARP, in the absence of cleavage product, was noted in cells treated with camptothecin only. This may be due to an inhibition of transcription. Apoptosis in cells treated with camptothecin plus anti-Fas could be completely blocked by pretreatment with ZVAD (50 μM) (Figure 5B). Thus, camptothecin sensitization involves the activation of caspases analogous to apoptosis in Fas-sensitive cells.

### Mitochondrial depolarization is a late and non-specific event in DU145 cells undergoing camptothecin-mediated anti-Fas-induced apoptosis

Emerging evidence suggests a key role for mitochondria during apoptosis induced by a diverse range of stimuli. Discontinuity of the outer mitochondrial membrane results, in some instances, in redistribution of cytochrome *c* from the intermembrane space to the cytosol, followed by subsequent inner mitochondrial membrane depolarization (Kroemer, 1997). Disruption of the transmembrane potential ($\Delta\psi m$) and generation of reactive oxygen intermediates (ROI) have been reported as early events in apoptosis (Lennon et al, 1991; Kroemer, 1997). $\Delta\psi m$ depolarization was only significant (but not complete) at 24 h, when > 80% of cells were dead (Figure 6). $\Delta\psi m$ disruption was evidently a late event in our system. Taken together, results suggest that, although degradation of $\Delta\psi m$ might contribute to the apoptosis of DU145 cells, it is unlikely that camptothecin causes sensitization to anti-Fas through this mechanism.

### Camptothecin opens the Fas apoptotic pathway(s) in DU145 cells through an ROI-independent mechanism

Apoptotic cells are believed to be subject to some form of oxidative stress, as they accumulate oxidized proteins and lipids (Buttke and Sandstrom, 1994) and, in a number of cell lines, apoptosis induced by chemotherapeutic drugs is accompanied by rapid production of ROI (Lennon et al, 1991; McGowan et al, 1996; Gorman et al, 1997b). Furthermore, Fas-mediated cell death has been shown to be blocked by superoxide anion (Clément and Stamenkovic, 1996), suggesting that an imbalance in ROI levels may have a role to play in Fas-mediated cell death. The existence of a short-lived inhibitor protein of Fas signalling transduction pathway(s) has been widely suggested. However, since the protein synthesis inhibitor cycloheximide did not sensitize DU145 cells to anti-Fas-mediated apoptosis, we sought to investigate alternative mechanism(s) which did not directly involve proteins. We hypothesized that camptothecin could be opening the Fas pathway in DU145 cells by either

© Cancer Research Campaign 1999

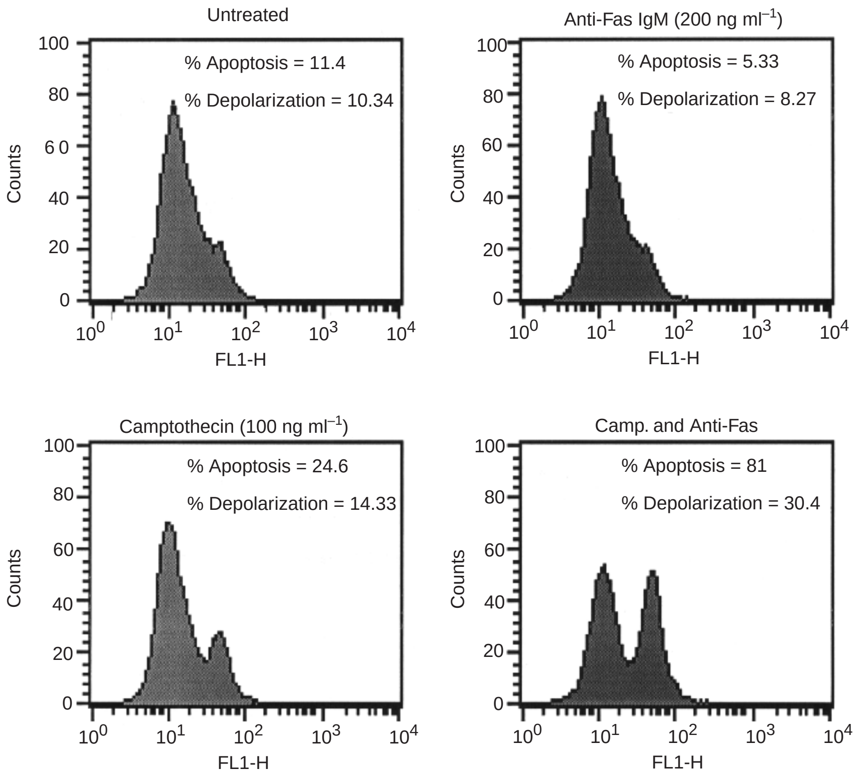

**Figure 6** Mitochondria transmembrane potential disruption in DU145 cells. In cells treated with camptothecin (100 ng ml$^{-1}$), anti-Fas IgM (200 ng ml$^{-1}$), or both, $\Delta\psi$m depolarization is a late event and does not correlate with apoptosis levels. FL-2H fluorescence of cells treated with camptothecin, anti-Fas, or both remained unchanged and was identical to the FL-2H of untreated cells. $\Delta\psi$m was measured by flow cytometry as described in Materials and Methods. Data are for the 24 h time point and are representative of three independent experiments increasing peroxide levels (Figure 7A) or by directly or indirectly causing a fall in superoxide levels (Figure 7B). None of the treatment (anti-Fas, camptothecin, camptothecin plus anti-Fas), under our experimental conditions, caused a significant increase in peroxide or a decrease in superoxide anion levels, as measured by flow cytometry. Camptothecin, at higher concentrations (1–10 μg ml$^{-1}$), did cause a rapid and marked increase in ROI levels (results not shown), but not at the concentrations used here (100 ng ml$^{-1}$, 24 h treatment). While it is possible that an alternative mechanism of oxidative stress contributes to camptothecin and anti-Fas cytotoxicities, the data presented in this study suggest that ROI are not significant players in camptothecin-mediated sensitization of DU145 cells to anti-Fas-induced apoptosis.

### DISCUSSION

Although prostate cancer cells expressed both Fas and FasL, they were significantly resistant to Fas-induced cell death. This observation, and the fact that the Fas/FasL system is emerging as one of the key apoptotic pathways in eukaryotic cells, prompted us to investigate why Fas-mediated apoptotic pathway(s) were blocked in these cells. Understanding the mechanisms underlying this resistance may allow the development of strategies for effectively killing prostate cancer cells, using a combination of cytotoxic and immune approaches. In this study, two prostate cancer cell lines (LNCaP, DU145) were used. It was shown that LNCaP and DU145 cells expressed both Fas and FasL. Treatment of LNCaP and DU145 with anti-Fas IgM and CHX up to 24 h induced moderate levels of apoptosis in LNCaP, having no significant cytotoxic effects on DU145 cells. LNCaP were more susceptible to anti-Fas when pretreated with CHX, suggesting that there might be a short-lived inhibitor in these cells. The existence of a hypothetical suppressor of Fas-induced apoptosis ('Saf' – suppressor of apoptosis by Fas) has been postulated (Green, 1997). Such suppressor is likely to exist, as in many cells resistance to Fas can be overcome by treatment with cyclohexamide or actinomycin D, which are thought to eliminate such short-lived inhibitor. FLIP (for Flice inhibitory protein), a molecule recently identified by Irmler and co-workers (1997), seems to be an appropriate candidate for such a role. A number of cytotoxic drugs were shown to sensitize androgen-independent cells (DU145) to anti-Fas-induced apoptosis. Indeed, DU145 cells were sensitized by ~20-fold when treated with subtoxic doses of camptothecin and concomitantly incubated with anti-Fas. This sensitization was not particular to topoisomerase I inhibitors, as actinomycin D, CPDD and etoposide also sensitized cells to cell death. Interestingly, we were unable to chemosensitize the androgen-responsive cells (LNCaP) using the above mentioned cytotoxic drugs.

It has been postulated that chemotherapeutic drugs induced apoptosis by activating the Fas/FasL apoptotic pathway (Friesen et al, 1996; Müller et al, 1997). There is an accumulating body of evidence which suggests that this is not always the case (Eischen et al, 1997; Villunger et al, 1997; McGahon et al, 1998). It has been suggested that lack of p53 expression might account for the lack of recruitment of Fas and FasL (Müller et al, 1997). Treatment of DU145 cells with camptothecin did not alter the levels of Fas, or

© Cancer Research Campaign 1999

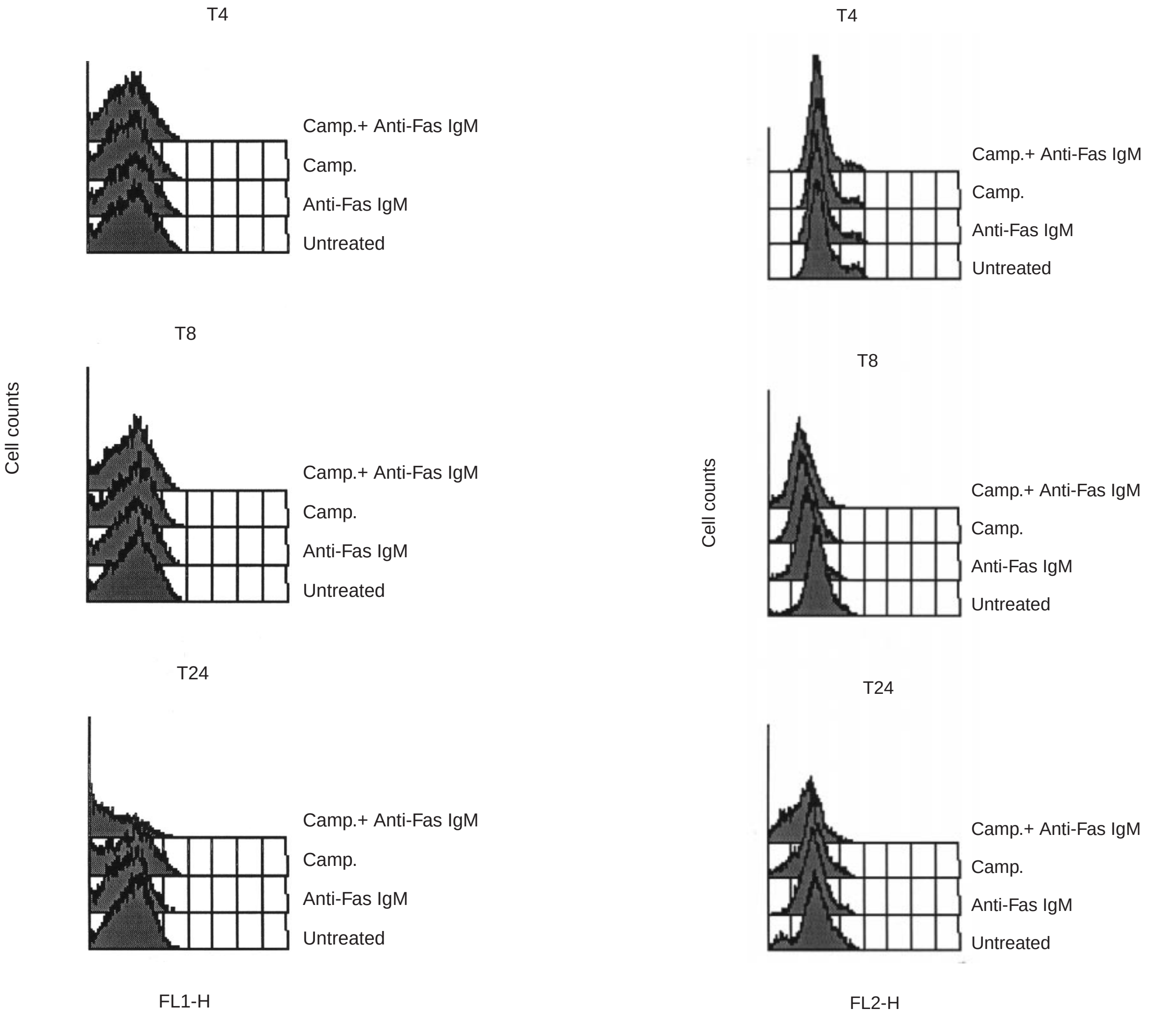

**Figure 7** (**A**) Camptothecin-mediated anti-Fas-induced apoptosis is independent of production of peroxide. Cells were treated with camptothecin (100 ng ml$^{-1}$), anti-Fas IgM (200 ng ml$^{-1}$), or both, and peroxide levels were assessed, as described in Materials and Methods. Data shown are for the 4, 8 and 24 h time point and are similar to those seen for earlier time points (0.5, 1 and 2 h). Data shown is representative of three independent experiments. (**B**) Levels of superoxide anion remained unaltered in DU145 cells undergoing camptothecin-mediated anti-Fas-induced apoptosis. Cells were treated with camptothecin (100 ng ml$^{-1}$), anti-Fas IgM (200 ng ml$^{-1}$), or both, and superoxide anion was as described in Materials and Methods. Data shown are for the 4, 8 and 24 h time points and are similar to those seen for 0.5, 1 and 2 h time points. Data shown is representative of three independent experiments

FasL, as shown by flow cytometry. DU145 cells have been shown to possess a mutated p53 (Isaacs et al, 1991), which further suggests that DU145 cells might be unable to up-regulate Fas or FasL proteins. In addition, cell cycle analysis of cells treated with anti-Fas, camptothecin, or camptothecin plus anti-Fas, has shown that camptothecin was mediating Fas-induced cell death through a mechanism which was independent of cell cycle alterations. Camptothecin has been shown to induce single-strand DNA breaks in the presence of topoisomerase I, which is a major target for the anti-tumour effect (Hsiang et al, 1989). How this is translated into a cytotoxic effect is, however, currently unknown.

Camptothecin sensitization was shown to involve activation of caspases analogous to apoptosis in Fas-sensitive cells. Indeed, poly(ADP-ribose) polymerase was proteolytically cleaved in cells treated with camptothecin plus anti-Fas, but not in cells treated with anti-Fas only. Furthermore, camptothecin-mediated sensitization of DU145 cells to Fas-induced apoptosis was completely blocked by the caspase inhibitor ZVAD.

A variety of stimuli have been shown to cause an accumulation of cytochrome *c* in the cytosol, cause disruption of mitochondria transmembrane potential and an increase in ROI, leading to activation of caspases, PARP cleavage, DNA degradation and subsequent apoptosis. We hypothesized that camptothecin could be sensitizing DU145 cells to anti-Fas-induced apoptosis through an ROI-mediated mechanism. Results, however, seem to negate this hypothesis: (1) an increase in peroxide, which is thought to induce apoptosis by increasing cellular oxidative stress beyond the tolerable threshold, could not be detected; (2) no decrease in superoxide anion was observed.

In summary, our results show that a combinatory treatment of androgen-independent DU145 cells with sublethal concentrations of camptothecin and very low concentrations of anti-Fas IgM, will kill nearly all cells (75–95%) in 24 h. The mechanism of this cooperation appears to be independent of alterations in the levels ROI (peroxide and superoxide anion). Because we were able to chemosensitize the androgen-independent cells (DU145), but not

*© Cancer Research Campaign 1999*

the androgen-responsive cells (LNCaP), it is tempting to speculate that the resistance of a prostate cell line to anti-Fas IgM treatment may be inversely proportional to its positive response to chemosensitization. This would have important implications for therapy since, although nearly all men with metastatic prostate cancer respond well initially to some sort of hormonal ablation therapy, prostate cancer cells invariably become androgen-independent. This is invariably fatal because no effective systemic therapy currently exists. Taken together, these results seem to indicate that a combination of chemotherapy and immunotherapy may present a novel approach for the elimination of androgen-independent cells.

### ACKNOWLEDGEMENTS

This work has been supported by grant PRAXIS XXI/BD/5857/95 from the Foundation for Science and Technology (Fundação para a Ciência e a Tecnologia), Lisbon, Portugal. The authors would like to thank Dr Sharon L McKenna for many helpful discussions.

### REFERENCES

Buttke TM and Sandstrom PA (1994) Oxidative stress as a mediator of apoptosis. *Immunol Today* **15**: 7–10

Carter HB and Coffey DS (1990) The prostate: an increasing medical problem. *Prostate* **16**: 187–197

Chung LWK (1995) The role of stromal-epithelial interaction in normal and malignant growth. *Cancer Surv* **23**: 33–42

Clément M-V and Stamenkovic I (1996) Superoxide anion is a natural inhibitor of Fas-mediated cell death. *EMBO J* **15**: 216–225

Costa-Pereira AP and Cotter TG (1999) Metabolic alterations associated with apoptosis. In *Apoptosis – a Practical Approach*, Studinzski G (ed). Oxford University Press: Oxford

Denmeade SR, Lin XS and Isaacs JT (1996) Role of programmed (apoptotic) cell death during the progression and therapy for prostate cancer. *Prostate* **28**: 251–265

Dobbelsteen DJ van den, Nobel CSI, Schlegel J, Cotgreave IA, Orrenius S and Slater AFG (1996) Rapid and specific efflux of reduced glutathione during apoptosis induced by anti-Fas/APO-1 antibody. *J Biol Chem* **271**: 15420–15427

Eischen CM, Kottke TJ, Martins LM, Basi GS, Tung JS, Earnshaw WC, Leibson PJ and Kaufmann SH (1997) Comparison of apoptosis in wild-type and Fas-resistant cells: chemotherapy-induced apoptosis is not dependent on Fas/Fas ligand interactions. *Blood* **90**: 935–943

Enari M, Talanian RV, Wong WW and Nagata S (1996) Sequential activation of ICE-like and CPP32-like proteases during Fas-mediated apoptosis. *Nature (Lond)* **380**: 723–726

Figueroa JA, Lee AV, Jackson JG and Yee D (1995) Proliferation of cultured human prostate cancer cells is inhibited by insulin-like growth factor (IGF) binding protein-1: evidence for an IGF-II autocrine growth loop. *J Clin Endocrinol Metab* **80**: 3476–3482

Friesen C, Herr I, Krammer PH and Debatin K-M (1996) Involvement of the CD95 (APO-1/Fas) receptor/ligand system in drug-induced apoptosis in leukaemia cells. *Nature Med* **2**: 574–577

Gorman AM, Samali A, McGowan AJ and Cotter TG (1997*a*) The use of flow cytometry techniques in studying mechanisms of apoptosis in leukemic cells. *Cytometry* **29**: 97–105

Gorman AM, McGowan AJ and Cotter TG (1997*b*) Role of peroxide and superoxide anion during tumour cell apoptosis. *FEBS* **404**: 27–33

Green DR (1997) A *Myc*-induced apoptosis pathway surfaces. *Science* **278**: 1246–1247

Herr I, Wilhelm D, Böhler T, Angel P and Debatin K-M (1997) Activation of CD95 (APO-1/Fas) signalling by ceramide mediates cancer therapy-induced apoptosis. *EMBO J* **16**: 6200–6208

Hsiang YH, Likan MG and Liu LF (1989) Arrest of replication forks by drug stabilised topoisomerase-I-DNA cleavable complexes as a mechanism of cell killing by camptothecin. *Cancer Res* **49**: 5077–5082

Huggins C and Hodges CV (1941) Studies on prostate cancer. I. The effect of castration, of oestrogen, and androgen injection on serum phosphatases in metastatic carcinoma of the prostate. *Cancer Res* **1**: 293–297

Irmler M, Thome M, Hahne M, Schneider P, Hofmann K, Steiner V, Bodmer J-L, Schröter M, Burns K, Mattmann C, Rimoldi D, French LE and Tschopp J (1997) Inhibition of death receptor signals by cellular FLIP. *Nature (Lond)* **388**: 190–195

Isaacs WB, Carter BS and Ewing CM (1991) Wild-type p53 suppresses growth of human prostate cancer cells containing mutant p53 alleles. *Cancer Res* **51**: 4716–4720

Kasahara Y, Iwai K, Yachie A, Ohta K, Konno A, Seki H, Miyawaki T and Taniguchi N (1997) Involvement of reactive oxygen intermediates in spontaneous and CD95(Fas/APO-1)-mediated apoptosis of neutrophils. *Blood* **89**: 1748–1753

Kroemer G (1997) Mitochondrial implication in apoptosis. Towards an endosymbiont hypothesis of apoptosis in evolution. *Cell Death Differ* **4**: 443–456

Lennon SV, Martin SJ and Cotter TG (1991) Dose-dependent induction of apoptosis in human tumour cell lines by widely diverse stimuli. *Cell Prolif* **24**: 203–204

Longthorne VL and Williams GT (1997) Caspase activity is required for commitment to Fas-mediated apoptosis. *EMBO J* **16**: 3805–3812

McGahon AJ, Costa-Pereira AP, Daly L and Cotter TG (1998) Chemotherapeutic drug-induced apoptosis in human leukaemic cells is independent of the Fas (APO-1/CD95) receptor/ligand system. *Br J Haematol* **101**: 539–547

McGowan AJ, Fernandes RS, Verhaegen S and Cotter TG (1994) Zinc inhibits UV radiation-induced apoptosis but fails to prevent subsequent cell death. *Int J Radiat Biol* **66**: 343–349

McGowan AJ, Ruiz-Ruiz MC, Gorman AM, Lopez-Rivas A and Cotter TG (1996) Reactive oxygen intermediate(s) (ROI): common mediator(s) of poly(ADP-ribose polymerase (PARP) cleavage and apoptosis. *FEBS* **392**: 299–303

McGowan AJ, Bowie AG, O'Neill LAJ and Cotter TG (1998) The production of a reactive oxygen intermediate (ROI) during the induction of apoptosis induced by cytotoxic agents. *Exp Cell Res* **238**: 248–256

Müller M, Strand S, Hug H, Heinemann E-M, Walczak H, Hofmann WJ, Stremmel W, Krammer PH and Galle PR (1997) Drug-induced apoptosis in hepatoma cells is mediated by the CD95 (APO-1/Fas) receptor/ligand system and involves activation of wild-type p53. *J Clin Invest* **99**: 404–413

Nagata S (1997) Apoptosis by death factor. *Cell* **88**: 355–365

Oehm A, Berhmann I, Falk W, Pawlita M, Maier G, Klas G, Li-Weber CM, Richards S, Dhein J, Trauth BC, Ponsting H and Krammer PH (1992) Purification and molecular cloning of the APO-1 cell surface antigen, a member of the tumour necrosis factor/nerve growth factor receptor superfamily. Sequence identity with the Fas antigen. *J Biol Chem* **267**: 10709–10715

Owen-Shaub LB, Radinsky R, Kruzel E, Berry K and Yonehara S (1994) Anti-Fas on non-hematopoietic tumours: levels of Fas/APO-1 and bcl-2 are not predictive of biological responsiveness. *Cancer Res* **54**: 1580–1586

Rokhlin OW, Bishop GA, Hostager BS, Waldschmidt TJ, Sidorenko SP, Pavloff N, Kiefer MC, Umanski SR, Glover RA and Cohen MB (1997) Fas-mediated apoptosis in human prostatic carcinoma cell lines. *Cancer Res* **57**: 1758–1768

Schulze-Osthoff K, Krammer P and Droge W (1994) Divergent signalling via APO-1/Fas and the TNF receptor, two homologues molecules involved in physiological cell death. *EMBO J* **13**: 4587–4596

Villunger A, Egle A, Kos M, Hartmann BL, Geley S, Kofler R and Greil R (1997) Drug-induced apoptosis is associated with enhanced Fas (Apo-1/CD95) ligand expression but occurs independently of Fas (Apo-1/CD95) signalling in human T-acute lymphatic leukemia cells. *Cancer Res* **57**: 3331–3334

Yagoda A and Petrylak D (1992) Cytotoxic chemotherapy for advanced hormone-resistant prostate cancer. *Cancer* **70**: 329–334

Yonehara S, Ishi A and Yonehara M (1989) A cell-killing monoclonal antibody (anti-Fas) to a surface antigen codownregulated with the receptor of tumour necrosis factor. *J Exp Med* **181**: 1747–1756

Widmann C, Gibson S and Johnson GL (1998) Caspase-dependent cleavage of signaling proteins during apoptosis. A turn-off mechanism for anti-apoptotic signals. *J Biol Chem* **273**: 7141–7147

Wyllie AH, Kerr JFR and Currie AR (1980) Cell death: the significance of apoptosis. *Int Rev Cytol* **68**: 251–306

 © Cancer Research Campaign 1999
